# Scleral Fixation of Akreos AO60 Intraocular Lens Using Gore-Tex Suture: An Eye on Visual Outcomes and Postoperative Complications

**DOI:** 10.1155/2021/9349323

**Published:** 2021-12-20

**Authors:** Mariana Leuzinger-Dias, Mário Lima-Fontes, Rita Rodrigues, Cláudia Oliveira-Ferreira, Carolina Madeira, Fernando Falcão-Reis, Vítor Fernandes, Amândio Rocha-Sousa, Manuel Falcão

**Affiliations:** ^1^Department of Ophthalmology, Centro Hospitalar e Universitário de São João, Porto, Portugal; ^2^Department of Surgery and Physiology, Faculty of Medicine of University of Porto, Porto, Portugal

## Abstract

**Purpose:**

“In-the-bag” placement of an IOL is the Holy Grail for any cataract surgeon. However, in the absence of capsular integrity, alternative surgical options to place the IOL must be sought. We aim to report the clinical outcomes and safety profile of scleral-fixated Akreos AO60 intraocular lens implantation using Gore-Tex suture, combined with pars plana vitrectomy.

**Methods:**

This is a single-center, retrospective case series descriptive study. Electronic clinical records of all patients subjected to scleral fixation of a Bausch and Lomb Akreos AO60 IOL combined with pars plana vitrectomy, between April 1, 2017, and August 1, 2021, were reviewed. Data concerning age, sex, laterality, past ophthalmological history, pre- and postoperative best-available visual acuity, surgical indication, and intra- and postoperative complications were collected. Measured outcomes were the differences in best-available visual acuity and frequency of postoperative complications.

**Results:**

A total of 37 eyes (20 right eyes and 17 left eyes) from 36 patients (16 females and 20 males) were included in the statistical analysis. The mean age at time of surgery was 72.0 ± 12.4 years. The mean follow-up period was 548.9 days (range 39–1564 days). Globally, the mean best-available logMAR visual acuity improved from 1.61 preoperatively (0.025 decimal equivalent) to 0.57 postoperatively (0.3 decimal equivalent), this difference being statistically significant (*P* < 0.001). Indications for surgery included aphakia due to complicated cataract surgery (24.3%; *n* = 9); subluxated IOL due to closed trauma (21.6%; *n* = 8); PEX-related subluxated IOL (16.2%; *n* = 6); non-traumatic, non-PEX-related subluxated IOL (18.9%; *n* = 7); subluxated crystalline lens due to closed trauma (8.1%; *n* = 3); aphakia due to open-globe injury (5.4%; *n* = 2); silicone-induced IOL opacification (2.7%; *n* = 1); and aphakia post-endophthalmitis (2.7%; *n* = 1). Postoperative complications included transient ocular hypertension (27.0%; *n* = 10), transient corneal edema (18.9%; *n* = 7), cystoid macular edema (18.9%, *n* = 7), self-limited hypotension (5.4%, *n* = 2), self-limited vitreous hemorrhage (2.7%, *n* = 1), central retinal vein occlusion (2.7%, *n* = 1), late retinal detachment (2.7%, *n* = 1), and Akreos IOL opacification (2.7%, *n* = 1). No suture-related complications were observed.

**Conclusion:**

There was a statistically significant improvement in visual acuity after scleral fixation of Akreos AO60 intraocular lens using Gore-Tex suture, with no suture-related problems recorded. This procedure seems to be a valuable alternative for posterior chamber IOL placement when secondary IOL implantation is required.

## 1. Introduction

Currently, “in-the-bag” placement of an intraocular lens (IOL) is the Holy Grail for any cataract surgeon. In such ideal conditions, the IOL is safely held and perfectly aligned with the pupillary axis, increasing the odds of best surgical and visual outcomes [1].

When posterior capsular integrity is disturbed, as it happens in complicated cataract surgery, but anterior capsular support is available, IOLs can be placed in the ciliary sulcus with satisfactory refractive outcomes [1].

However, in a wide range of conditions, namely, those predisposing to zonular fragility (connective tissue diseases, pseudoexfoliation syndrome, homocystinuria, and so on), ocular trauma, or zonular damage during complicated cataract surgery, both anterior and posterior capsules are compromised, hampering classical “in-the-bag” or sulcus positioning. In this scenario, aphakia can be managed by the implantation of an anterior chamber intraocular lens (ACIOL), iris-fixated intraocular lens (IFIOL), or scleral-fixated intraocular lens (SFIOL) [1–3]. In the past, all these types of IOLs were non-foldable, thus requiring large corneal incisions for intraocular placement. Each of these techniques has its pros and cons, and none has proved to be superior to the other [4]. Thus, the choice of the best surgical modality depends on surgeon preference and experience, patient characteristics, eye anatomy, and ocular comorbidities [1, 2]. SFIOL may be valuable in cases where there is an increased risk of corneal endothelial cell loss, disqualifying an anterior chamber IOL [3]. Besides, by placing the IOL in the posterior chamber in a more physiological location, this surgical modality may potentially offer greater refractive advantages [5]. Similarly, retropupillary iris-claw IOLs could also offer such an advantageous physiological position and lower risk of endothelial cell loss [6]. However, iris structure does not always allow for IFIOL placement [7], especially in eyes that underwent trauma or complicated surgery, as it frequently is the case. Moreover, the correct enclavation of this type of IOL is a highly demanding surgical maneuver with a long learning curve [8]. Recently, a new technique using a guide needle to facilitate the enclavation was proposed by Frisina et al. with promising results [8]. Still, in either case, placing a posterior chamber iris-claw IOL usually requires large corneal incisions when compared to foldable SFIOL positioning, potentially inducing greater corneal astigmatism.

Nevertheless, SFIOL implantation is not without its drawbacks. Suture degradation and knot-related complications are chief concerns with this surgical approach. Vote and colleagues reported a proportion of suture breakage of 27.9%, with the traditionally used 10–0 polypropylene sutures [9]. Larger diameter 9–0 polypropylene sutures are theoretically more resistant, but a 2.7% rate of suture breakage is still non-neglectable considering the associated risk of sight-threatening endophthalmitis [10]. Sutureless scleral fixation techniques have been proposed with centered posterior chamber IOL positioning. These techniques may potentially solve suture-related complications, but problems associated with haptics slippage and subsequent IOL dislocation remain as important complications [1, 7, 11]. Recently, new foldable IOLs such as the Carlevale IOL have been designed for scleral fixation, allowing small incision sutureless implantation with great IOL stability and promising results [11]. In specific cases, this technique can be safely combined with other surgical procedures, as demonstrated by Kymionis et al. who performed a combined DSAEK with the scleral implantation of a Carlevale IOL in a patient with bullous keratopathy and a dislocated IOL [12]. Still, high intraocular pressure (IOP), cystoid macular edema, and iris capture by the IOL optic are reported complications with sutureless scleral fixation IOLs [11].

Classically used in heart valve and vascular surgeries, Gore-Tex is a non-absorbable, polytetrafluoroethylene monofilament suture that has recently assumed a relevant role in scleral IOL fixation due to its superior tensile strength and supposed greater resiliency, when compared to polypropylene [3]. Besides its increased durability, Gore-Tex suture is easy to control due to its reduced memory and does not induce any inflammatory response, and thanks to its white color, it is clearly distinguishable from the background tissues [3]. More importantly, there are no Gore-Tex suture breakage reports published in the literature so far.

Different types of IOLs can be scleral fixated. Alcon CZ70BD and Bausch and Lomb Akreos AO60 are two of the most used ones. The first is a non-foldable lens and has a single eyelet on each side of the optic center, and its implantation requires the construction of a scleral tunnel. The latter has two eyelets on each side of the optic center, is foldable along its axis, and is currently used off-label for this technique [3, 13].

In this study, we describe the clinical outcomes and safety profile of scleral-fixated Akreos AO60 intraocular lens implantation using Gore-Tex suture, combined with pars plana vitrectomy (PPV), performed in a Portuguese tertiary hospital. To the best of our knowledge, this is the first European study of its kind.

## 2. Materials and Methods

### 2.1. Study Design and Population

This is a single-center, retrospective case series descriptive study. Electronic clinical records of all patients subjected to scleral fixation of a Bausch and Lomb Akreos AO60 IOL combined with PPV at the ophthalmology department of Centro Hospitalar Universitário de São João between 1 April 2017 and 1 August 2021 were reviewed. The patients were selected from surgical reports for corresponding procedural codification. A total of 42 eyes of 41 patients were identified. Of these, 5 patients were excluded for a follow-up period inferior to 1 month, and 37 eyes from 36 patients were included in the statistical analysis. The study was developed in accordance with the tenets of the Declaration of Helsinki.

### 2.2. Data Collection and Definitions

Data concerning age, sex, laterality, past ophthalmological history, pre- and postoperative best-available visual acuity (VA), surgical indication, and intra- and postoperative complications were collected. Considered outcomes were the differences in best-available VA and frequency of intra- and postoperative complications. Visual acuity was measured by the distance Snellen chart preoperatively and at the last postoperative visit. IOP was measured by Goldmann applanation tonometry. Hypotony was defined as intraocular pressure (IOP) of 5 mmHg or less, and hypertension was defined as an IOP of 25 mmHg or more, at any postoperative visit, following previous similar definitions in other studies [3, 12, 13]. Corneal edema was defined as de novo postoperative edema persisting for more than 1 week. Cystoid macular edema was defined by the presence of de novo macular cysts, confirmed by spectral-domain optical coherence tomography (SD-OCT), performed by the first postoperative month. Visual acuities were converted from decimal to the logarithm of the minimum angle of resolution (logMAR) equivalents for statistical analysis. As in similar studies, a VA of counting fingers and hand motions was transformed to a logMAR of 1.98 and 2.28, respectively [13].

### 2.3. Statistical Analysis

Statistical analysis was performed using the IBM^®^ SPSS^®^ Statistics software (version 27.0 for Windows; SPSS Inc., Chicago, IL, USA). Variables' normal distribution was verified by skewness, kurtosis, and Kolmogorov–Smirnov test. Parametric or non-parametric tests were used for variables comparison, according to the data distribution. The level of significance was established at a *P* value of <0.05.

### 2.4. Surgical Technique

**Figure 1:**
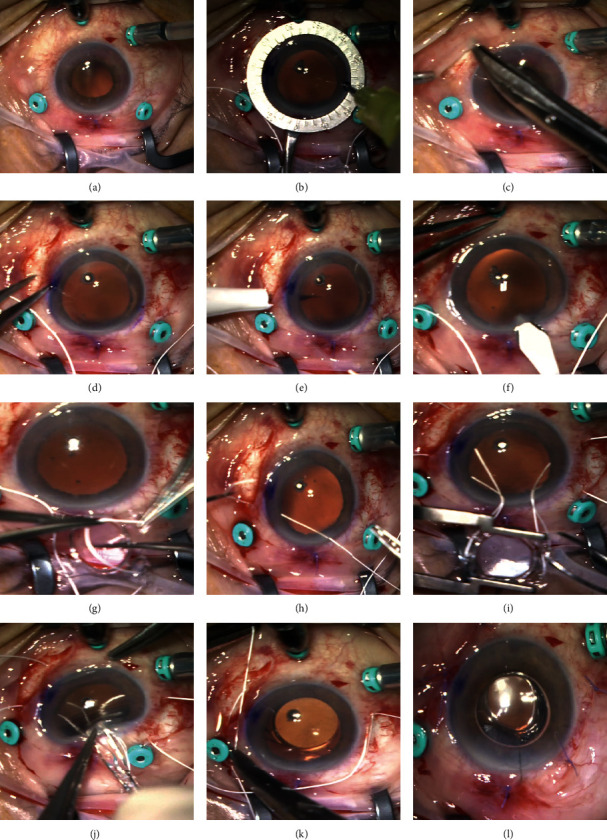
Illustration of basic surgical steps. The procedure begins with a standard 25-gauge three-port PPV. Here an inferior chandelier was also used (a). A Mendez ring is used to mark the horizontal axis to assure adequate sclerotomy positioning and lens centration (b), and nasal and temporal limited conjunctival peritomies are created (c). Calipers are used to mark the sclerotomy sites 2.5 mm behind the limbus and 5 mm apart (d), and four distinct sclerotomies (two nasal and two temporal) are made using the 25-gauge empty trocar needle (e). A 3.2 mm clear corneal incision is then made (f). The Gore-Tex suture is cut in half, the needle is removed, and the suture is then looped through the eyelets of the IOL (g). Each end of the suture is then transferred to the anterior chamber externalized through the corresponding sclerotomy, using non-serrated vitrectomy forceps (h). The Akreos IOL is easily folded and fits through the 3.2 mm corneal incision (i, j). The knots are tied with a 3-1-1 technique and rotated into the sclerotomy (k). Conjunctival peritomies are carefully closed with 7–0 vicryl. The corneal incision is usually self-sealing. Here a 10–0 monofilament suture was required (l).

The basic steps of this technique are represented in Figure 1. The procedure begins with a standard three-port PPV, performed with 25-gauge vitrectomy trocars. A toric lens marker, usually a Mendez ring, is then used to mark the corneal limbus at 2 different sites, 180° apart. Nasal and temporal conjunctival peritomies are created with an approximate extension of 6 mm. Then, four distinct sclerotomies (two nasal and two temporal) are made using either the 23 or 25-gauge empty trocar needle, 2.5 mm behind the limbus, 5 mm apart and centered around the horizontal axis. A superior corneal incision is done with a 3.2 mm keratome knife. Outside the eye, the 7–0 Gore-Tex suture (cut in half and with its needle removed) is passed through each pair of eyelets of the Akreos AO60 IOL, in a “U-shaped” configuration. Then, each end of the suture is transferred to the anterior chamber via the corneal incision and subsequently externalized through the corresponding sclerotomy, using non-serrated vitrectomy forceps. Afterward, the Akreos AO60 IOL is folded along its longer axis and introduced inside the eye. The Gore-Tex sutures are tied, and the knot is buried into the sclerotomy. Suture tension adjustments are made to assure that the lens is perfectly centered and adequately positioned in the posterior chamber. Finally, conjunctival peritomies are carefully closed with 7–0 vicryl. The corneal main port is usually self-sealing, but sometimes leaky incisions require a 10–0 monofilament suture.

This standard surgical technique can suffer mild modifications based on accumulated experience and the surgeon's preferences. For example, Akreos AO60 IOL can be inserted into the anterior chamber loaded in an injector, with the Gore-Tex suture being passed through the lens eyelets in the anterior chamber. This allows for a smaller corneal incision.

The surgeries were performed by three different surgeons.

IOL power was determined by traditional biometry, based on an “in-the-bag” calculation.

## 3. Results

### 3.1. Baseline Characteristics

A total of 42 eyes of 41 patients were identified. Of these, 37 eyes (20 right eyes and 17 left eyes) from 36 patients had a minimum of 1 month of follow-up and were included in the statistical analysis. 44.4% (*n* = 16) of patients were females. The mean age at the time of surgery was 72.0 ± 12.4 years old (range 31–92 years old). The mean follow-up period was 548.9 days (range 39–1564 days).

Relevant past ophthalmological history included closed-globe trauma in 12 eyes (32.4%), pseudoexfoliation (PEX) syndrome in 8 eyes (21.6%), glaucoma in 7 eyes (18.9%), previous PPV for retinal detachment repair in 5 eyes (13.5%), pathological myopia in 4 eyes (10.8%), open-globe trauma in 3 eyes (8.1%), exudative age-related macular degeneration in 2 eyes (5.4%), diabetic retinopathy without macular edema in 2 eyes (5.4%), dry age-related macular degeneration, previous penetrating keratoplasty, endophthalmitis, diabetic retinopathy with macular edema, retinal venous occlusion, toxic optic neuropathy, and Vogt–Koyanagi–Harada syndrome in 1 eye each (2.7%). Population baseline characteristics are represented in Table 1.

Indications for surgery included aphakia due to complicated cataract surgery (24.3%; *n* = 9); subluxated IOL due to closed trauma (21.6%; *n* = 8); non-traumatic, non-PEX-related subluxated IOL (18.9%; *n* = 7); PEX-related subluxated IOL (16.2%; *n* = 6); subluxated crystalline lens due to closed trauma (8.1%; *n* = 3); aphakia due to open-globe injury (5.4%; *n* = 2); silicone-induced IOL opacification (2.7%; *n* = 1); and aphakia post-endophthalmitis (2.7%; *n* = 1).

All patients underwent scleral fixation of an Akreos AO60 IOL using Gore-Tex suture, combined with either 23- or 25-gauge PPV. 1 eye (2.7%) underwent concomitant glaucoma surgery with Ahmed valve implantation.

### 3.2. Visual Outcomes

The mean best-available preoperative logMAR VA was 1.61 ± 0.73 (0.025 decimal equivalent). The mean best-available postoperative logMAR VA was 0.57 ± 0.66 (0.3 decimal equivalent), and, globally, the improvement from pre- to postoperative best-available VA was statistically significant (*P* < 0.001). VA was 5/10 (logMAR 0.3) or better in 3 eyes (8.1%) preoperatively, as compared to 17 eyes (45.9%) postoperatively. Subgroup analysis considering indication for surgery revealed a statistically significant postoperative vision improvement for patients with aphakia due to complicated cataract surgery (*P*=0.028), subluxated IOL due to closed trauma (*P*=0.028), non-traumatic, non-PEX-related subluxated IOL (*P*=0.028), and PEX-related subluxated IOL (*P*=0.043). Visual improvement was noted for patients in the remaining subgroups, but this difference did not reach statistical significance. Subgroups of silicone-induced IOL opacification and aphakia post-endophthalmitis included a single eye, and statistical significance could not be calculated.

During the study period, 1 eye (2.7%) had postoperative visual deterioration of 2 lines in the Snellen chart, and 7 eyes (21.6%) had no change in VA.

### 3.3. Intraoperative Complications

There were 3 eyes (8.1%) with reported intraoperative complications: one iatrogenic retinal hole done during vitrectomy; a flat peripherical serous choroidal detachment; and an intraoperative vitreous hemorrhage.

### 3.4. Postoperative Complications

Postoperative complications included ocular hypertension (27.0%; *n* = 10), transient corneal edema (18.9%; *n* = 7), cystoid macular edema (18.9%, *n* = 7), self-limited hypotension (5.4%, *n* = 2), self-limited vitreous hemorrhage (2.7%, *n* = 1), one case of central retinal vein occlusion (2.7%), one case of late retinal detachment (2.7%), and one case of Akreos IOL opacification (2.7%). Retinal detachment was managed with PPV with gas endotamponade; Akreos opacification is awaiting surgery to replace the IOL; the central retinal vein occlusion has been receiving intravitreal injections of 1.25 mg bevacizumab and 2 cases of macular edema resolved after intravitreal injections of corticosteroids (one case with intravitreal 2 mg triamcinolone alone, and the other with 2 mg triamcinolone, followed by 0.7 mg dexamethasone intravitreal implant). All of the other complications were managed medically, with topical treatment. No suture-related complications were observed, namely, suture breakage, IOL displacements, or suture-related inflammation. Also, there were no cases of postoperative endophthalmitis, choroidal detachment, or uveitis-glaucoma-hyphema syndrome.

Clinical outcomes are reviewed in Table 2.

## 4. Discussion

In the absence of capsular or iris support secondary to ocular trauma, zonular weakness, or complicated cataract surgery, scleral-sutured IOLs are a viable option in the surgical management of aphakia. Since its first description in the ‘80s by Malbran et al. [14], using a 10–0 polypropylene suture, the surgical technique and materials used have been evolving to improve its safety profile and success rate. Known by the resilience shown in non-ophthalmic surgery, Gore-Tex sutures have been recently used off-label in the scleral fixation of an IOL, to deal with suture-related complications associated with polypropylene. Although the theoretical benefits of Gore-Tex are obvious, long-term studies are critical to proving its practical effectiveness.

To the best of our knowledge, this is the first European case series assessing the visual outcomes and safety profile of Gore-Tex suture in the scleral fixation of Akreos AO60 intraocular lens combined with PPV, with a mean follow-up period of 548.9 days.

In our series, globally, the mean best-available logMAR VA improved from 1.61 preoperatively to 0.57 postoperatively, and this difference was statistically significant. This agrees with visual improvement observed in previous studies [3, 13].

During the study period, one eye (2.7%) had a postoperative visual deterioration of 2 lines in the Snellen chart. In this specific case, the patient's preoperative best-available VA was 10/10, and the surgery was justified by a subluxated IOL leading to unbearable monocular diplopia, rather than to low VA. Postoperatively, his best-available VA was 8/10 and there was complete resolution of the diplopia complaints, and thus it was regarded as a successful outcome. Further, 7 eyes (21.6%) presented no change in VA. 6 of these had a very low preoperative vision, with a past ophthalmological history explaining the lack of visual improvement (exudative AMD with a disciform scar, toxic optic neuropathy, terminal glaucoma, open-globe injury, bullous keratopathy, and myopic macular scar), and one developed a central retinal vein occlusion, 3 weeks after surgery, hampering visual recuperation.

Intraoperative complications were reported in 3 eyes, but all of them were minor and non-sight threatening. The serous choroidal detachment was small, flat, and peripherical and resolved by the first 24 postoperative hours. The intraoperative vitreous hemorrhage was also self-limited and managed with simple observation. The iatrogenic retinal hole occurring during vitrectomy was successfully managed with endolaser circumscribing the lesion.

Our most common postoperative complication was ocular hypertension (27.0%; *n* = 10), defined as de novo IOP of 25 mmHg or more, at any postoperative visit. Of these patients, 8 presented with conditions that can possibly facilitate such rise in postoperative IOP: 3 had a history of glaucoma, 2 underwent surgery due to complicated cataract surgery with retained lens material, and 3 had a history of closed-globe trauma. All ten cases of ocular hypertension were successfully treated medically, with hypotensive drops.

In our series, transient hypotony (defined as de novo IOP of 5 mmHg or less, at any postoperative visit) occurred in 5.4% of cases (*n* = 2). This value is lower than what was previously described [3, 13]. Such a low rate might be explained by the predominant use of small gauge vitrectomy instrumentation (25-gauge) that diminishes leakage from sclerotomy sites.

Our rate of postoperative cystoid macular edema (18.9%, *n* = 7) was higher than that in other reports [3, 13]. Of these eyes, 3 underwent PPV with posterior phaco-fragmentation due to complicated cataract surgery with retained lens material; 2 had a history of PEX syndrome and the other 2 were diabetic patients (one without diabetic retinopathy and the other with minimal non-proliferative diabetic retinopathy). Such pro-inflammatory conditions might justify the development of this complication [15, 16]. All eyes were treated with topical nepafenac 3 mg/ml and dexamethasone 1 mg/ml drops. Two of those eyes were resistant to medical treatment, and edema resolution required intravitreal injections of corticosteroids (1 case with intravitreal 2 mg triamcinolone alone, and the other with 2 mg triamcinolone, followed by 0.7 mg dexamethasone intravitreal implant).

We reported a case (2.7%) of macula-on retinal detachment, occurring 5 months after surgery. This postoperative complication had never been reported in the literature so far with this technique. This patient underwent PPV with posterior phaco-fragmentation due to complicated cataract surgery with retained lens material and she had no relevant past ophthalmic history. Therefore, a cause-effect relationship is difficult to establish. Our 2.7% value is lower than that in previous studies with different techniques. Vote et al. reported an 8.2% rate of retinal detachment after combined PPV and scleral fixation of an Alcon CZ 70 BD IOL using 10–0 polypropylene suture [9], and Czajka et al. described a 3.8% rate of retinal detachment occurring after combined PPV and sutureless scleral fixation of a three-piece IOL [7].

**Figure 2:**
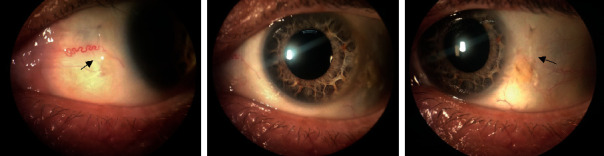
Slit-lamp photograph of a patient's left eye, 2 months after scleral fixation of an Akreos AO60 due to aphakia after complicated cataract surgery. The Gore-Tex suture (arrows) is barely visible underneath the conjunctiva, the knots are adequately buried into the sclerotomy, and there is no suture-related inflammatory reaction. The middle panel shows a perfectly centered IOL (courtesy of Dr. Sónia Torres-Costa).

No suture-related complications were observed, namely, suture breakage, IOL displacements, or suture-related inflammation (Figure 2), even in eyes with the longest follow-up period (1564 days). But additional long-term follow-up studies would be important to confirm the resilient profile of Gore-Tex sutures over time.

Recently, a foldable, single-piece, sutureless SFIOL called Carlevale has been introduced. Besides being devoid of suture-related complications, Carlevale IOL has been reported to have a great stability profile with no IOL displacement or haptic breakage, providing good refractive outcomes. Moreover, its innovative design with two small and flexible plugs at the end of each haptic, anchoring the IOL to the scleral tissue, theoretically reduces surgical complexity and time [11, 12].

A comparative analysis between scleral-fixated IOLs using Gore-Tex suture and this novel sutureless technique is lacking.

The advantages of Akreos AO60 IOL have been described elsewhere [2, 3, 13] and include its stability and an inferior chance of lens tilt and induced astigmatism, due to its two pairs of eyelets allowing for a 4-point scleral fixation, along with its ability to be easily folded and introduced in the eye through smaller corneal incisions. However, cases of optic opacification have been described with these hydrophilic lenses after both anterior and posterior segment procedures [17–19]. Indeed, we reported one case of IOL opacification, severely compromising VA. Considering that this was a late complication, occurring 2 years postoperatively, a longer follow-up period would be critical to understanding the real burden of this issue. Comparative studies, using different types of IOLs would help to determine the best IOL to implant.

In our series, all patients underwent scleral fixation of an Akreos AO60 IOL using Gore-Tex suture, combined with PPV. However, it is important to recognize that scleral-fixated IOLs can be placed without the need for concurrent PPV, with good results [20].

This study has some limitations, primarily related to its retrospective design.

Best-corrected VA determined after objective or/and subjective refraction was not always available, and the best-available VA with pinhole was considered in those cases. Therefore, an underestimation of the final visual outcomes might have occurred. Secondly, the surgeries were performed by three distinct surgeons with different surgical experience and preferences, introducing some variability to the standard technique, as was mentioned above, which could limit generalizability. Also, there was a large interval in follow-up duration, ranging from 39 to 1564 days. There were 21 eyes (56.8%) with a follow-up of less than 1 year. Thus, potential late complications in this group could not be assessed. Finally and importantly, there was a wide range of surgical indications and complex ophthalmological backgrounds that might confound the interpretation of the postoperative outcomes and complications. Although we believe that we have a real-world representative sample, a larger population would further increase the power of the results.

## 5. Conclusions

Our study reports a statistically significant improvement in VA after scleral fixation of Akreos AO60 intraocular lens using Gore-Tex suture, with no suture-related complications. This technique can be safely combined with PPV and can thus be a valuable option for posterior segment surgeons when both vitreoretinal surgery and secondary IOL implantation are required. In future, prospective studies with a longer follow-up period and a larger population would be important to prove the long-term safety profile of this procedure. Also, comparative analysis with other IOL types and with other treatment strategies for aphakia would be necessary to conclude the advantages of Gore-Tex scleral-fixated Akreos AO60 IOL over them.

## Figures and Tables

**Table 1:** Patient baseline characteristics.

Total patients, *n*	36

Total eyes, *n*	37
Right eye: left eye	20 : 17
Male: female, *n*	20 : 16
Age (years)	
Mean ± SD	72.0 ± 12.4
Range	31–92
Past ophthalmic history, *n* (%)	
Closed-globe trauma	12 (32.4%)
PEX syndrome	8 (21.6%)
Glaucoma	7 (18.9%)
Retinal detachment repair by PPV	5 (13.5%)
Pathological myopia	4 (10.8%)
Open-globe trauma	3 (8.1%)
Exudative AMD	2 (5.4%)
Diabetic retinopathy without DME	2 (5.4%)
Dry AMD	1 (2.7%)
Penetrating keratoplasty	1 (2.7%)
Retinal venous occlusion	1 (2.7%)
Toxic optic neuropathy	1 (2.7%)
VKH syndrome	1 (2.7%)
Surgical indication, *n* (%)	
Aphakia due to complicated cataract surgery	9 (24.3%)
Subluxated IOL due to closed trauma	8 (21.6%)
Non-traumatic, non-PEX-related subluxated IOL	7 (18.9%)
PEX-related subluxated IOL	6 (16.2%)
Subluxated crystalline lens due to closed trauma	3 (8.1%)
Aphakia due to open-globe injury	2 (5.4%)
Silicone-induced IOL opacification	1 (2.7%)
Aphakia post-endophthalmitis	1 (2.7%)
Follow-up period, days	
Mean	548.9
Range	39–1564

AMD, age-related macular degeneration; DME, diabetic macular edema; IOL, intraocular lens; PEX, pseudoexfoliation; PPV, pars plana vitrectomy; VA, visual acuity.

**Table 2:** Clinical outcomes.

*Visual acuity*			

	Preop. logMAR VA, mean ± SD	Postop. logMAR VA, mean ± SD	*P*
Overall (*n* = 37)	1.61 ± 0.73	0.57 ± 0.66	(*P* < 0.001)
Surgical indication (*n*)			
Aphakia due to complicated cataract surgery (9)	1.46 ± 0.68	0.51 ± 0.45	0.028
Subluxated IOL due to closed trauma (8)	1,65 ± 0.83	0.44 ± 0.76	0.028
Non-traumatic, non-PEX-related subluxated IOL (7)	1.61 ± 0.75	0.65 ± 0.66	0.028
PEX-related subluxated IOL (6)	1.47 ± 0.81	0.32 ± 0.25	0.043
Subluxated crystalline lens due to closed trauma (3)	2.08 ± 0.17	1.35 ± 1.08	0.317
Aphakia due to open-globe injury (2)	2.13 ± 0.21	1.09 ± 1.26	0.317
Silicone-induced IOL opacification (1)	0.15 ± 0.00	0.00 ± 0.00	†
Aphakia post-endophthalmitis (1)	2.28 ± 0.00	0.30 ± 0.00	†

*Intraoperative complications*			
	*n* (%)	Treatment	
Iatrogenic retinal hole	1 (2.7%)	Endolaser	
Choroidal detachment	1 (2.7%)	Observation	
Vitreous hemorrhage	1 (2.7%)	Observation	

*Postoperative complications*			
	*n* (%)	Treatment	
Ocular hypertension	10 (27.0%)	Topical	
Corneal edema	7 (18.9%)	Topical	
Cystoid macular edema	7 (18.9%)	Topical + intravitreal injections^*∗*^	
Hypotension	2 (5.4%)	Observation	
Vitreous hemorrhage	1 (2.7%)	Observation	
Central retinal vein occlusion	1 (2.7%)	Intravitreal injections	
Retinal detachment	1 (2.7%)	Surgical	
Akreos IOL opacification	1 (2.7%)	Surgical	

PEX, pseudoexfoliation. †This subgroup includes 1 case, and a *P* value is impossible to calculate. ^*∗*^Five eyes with macular edema responded to topical nepafenac 3 mg/ml + dexamethasone 1 mg/ml drops; 2 eyes were refractory to topical drops and needed intravitreal injections of corticosteroids (1 case with intravitreal 2 mg triamcinolone alone, and the other with 2 mg triamcinolone, followed by 0.7 mg dexamethasone intravitreal implant).

## Data Availability

The data used to support the findings of this study cannot be made publicly available, as no patient approval has been obtained for sharing coded data. Output of statistical analyses can be made available upon request.

## Supplementary Materials

Supplementary Materials The supplementary material includes a video showing the surgical technique in detail (https://drive.google.com/file/d/1-ae40KWv_w2TjBt-K193CqyrJ8ZaKx93/view?usp=sharing).

## References

[1] Stem M. S., Todorich B., Woodward M. A., Hsu J., Wolfe J. D. (2017). Scleral-fixated intraocular lenses: past and present. *Journal of VitreoRetinal Diseases*.

[2] Botsford B. W., Williams A. M., Conner I. P., Martel J. N., Eller A. W. (2019). Scleral fixation of intraocular lenses with gore-tex suture: refractive outcomes and comparison of lens power formulas. *Ophthalmology Retina*.

[3] Khan M. A., Gupta O. P., Smith R. G. (2016). Scleral fixation of intraocular lenses using Gore-Tex suture: clinical outcomes and safety profile. *British Journal of Ophthalmology*.

[4] Wagoner M. D., Cox T. A., Ariyasu R. G., Jacobs D. S., Karp C. L. (2003). Intraocular lens implantation in the absence of capsular support: a report by the American academy of ophthalmology. *Ophthalmology*.

[5] Khan M. A., Gerstenblith A. T., Dollin M. L., Gupta O. P., Spirn M. J. (2014). Scleral fixation of posterior chamber intraocular lenses using gore-tex suture with concurrent 23-gauge pars plana vitrectomy. *Retina*.

[6] Mora P., Calzetti G., Favilla S. (2018). Comparative analysis of the safety and functional outcomes of anterior versus retropupillary iris-claw IOL fixation. *Journal of ophthalmology*.

[7] Czajka M. P., Frajdenberg A., Stopa M., Pabin T., Johansson B., Jakobsson G. (2020). Sutureless intrascleral fixation using different three-piece posterior chamber intraocular lenses: a literature review of surgical techniques in cases of insufficient capsular support and a retrospective multicentre study. *Acta Ophthalmologica*.

[8] Frisina R., Pilotto E., Tozzi L., Parrozzani R., Midena E. (2019). A new technique of needle-guided retropupillary fixation of iris-claw intraocular lens. *Journal of Cataract & Refractive Surgery*.

[9] Vote B. J., Tranos P., Bunce C., Charteris D. G., Da Cruz L. (2006). Long-term outcome of combined pars plana vitrectomy and scleral fixated sutured posterior chamber intraocular lens implantation. *American Journal of Ophthalmology*.

[10] Obeng F. K., Vig V. K., Singh P., Singh R., Dhawan B., Sahajpal N. (2017). Posterior chamber scleral fixation of intraocular lenses in post-vitrectomised aphakic eyes. *Journal of Clinical and Diagnostic Research*.

[11] Fiore T., Messina M., Muzi A. (2021). Comparison of two different scleral fixation techniques of posterior chamber Carlevale lens. *Medicine*.

[12] Kymionis G., Petrelli M., Schmutz L., Petrovic A. (2020). New sutureless, scleral-fixated intraocular lens (Carlevale, soleko) implantation combined with descemet stripping automated endothelial keratoplasty: an innovative surgical approach. *Cornea*.

[13] Khan M. A., Samara W. A., Gerstenblith A. T. (2018). Combined pars plana vitrectomy and scleral fixation of an intraocular lens using gore-tex suture: one-year outcomes. *Retina*.

[14] Malbran E. S., Malbran E., Negri I. (1986). Lens guide suture for transport and fixation in secondary IOL implantation after intracapsular extraction. *International Ophthalmology*.

[15] Ilveskoski L., Taipale C., Holmström E. J., Tuuminen R. (2019). Macular edema after cataract surgery in eyes with and without pseudoexfoliation syndrome. *European Journal of Ophthalmology*.

[16] Wielders L. H. P., Schouten J. S. A. G., Winkens B. (2018). Randomized controlled European multicenter trial on the prevention of cystoid macular edema after cataract surgery in diabetics: ESCRS PREMED Study Report 2. *Journal of Cataract & Refractive Surgery*.

[17] Balendiran V., MacLean K., Mamalis N., Tetz M., Werner L. (2019). Localized calcification of hydrophilic acrylic intraocular lenses after posterior segment procedures. *Journal of Cataract & Refractive Surgery*.

[18] Kalevar A., Dollin M., Gupta R. R. (2020). Opacification of scleral-sutured akreos ao60 intraocular lens after vitrectomy with gas tamponade: case series. *Retinal Cases & Brief Reports*.

[19] Werner L., Wilbanks G., Nieuwendaal C. P. (2015). Localized opacification of hydrophilic acrylic intraocular lenses after procedures using intracameral injection of air or gas. *Journal of Cataract & Refractive Surgery*.

[20] Sindal M. D., Nakhwa C. P., Sengupta S. (2016). Comparison of sutured versus sutureless scleral-fixated intraocular lenses. *Journal of Cataract & Refractive Surgery*.

